# Morphology Development and Flow Characteristics during High Moisture Extrusion of a Plant-Based Meat Analogue

**DOI:** 10.3390/foods10081753

**Published:** 2021-07-29

**Authors:** Patrick Wittek, Felix Ellwanger, Heike P. Karbstein, M. Azad Emin

**Affiliations:** Institute of Process Engineering in Life Sciences, Chair of Food Process Engineering, Karlsruhe Institute of Technology, 76131 Karlsruhe, Germany; patrick.wittek@kit.edu (P.W.); felix.ellwanger@kit.edu (F.E.); heike.karbstein@kit.edu (H.P.K.)

**Keywords:** high moisture extrusion, meat analogue, plant protein, soy protein, anisotropic structures, morphology development, flow characteristics, computational fluid dynamics

## Abstract

Plant-based meat analogues that mimic the characteristic structure and texture of meat are becoming increasingly popular. They can be produced by means of high moisture extrusion (HME), in which protein-rich raw materials are subjected to thermomechanical stresses in the extruder at high water content (>40%) and then forced through a cooling die. The cooling die, or generally the die section, is known to have a large influence on the products’ anisotropic structures, which are determined by the morphology of the underlying multi-phase system. However, the morphology development in the process and its relationship with the flow characteristics are not yet well understood and, therefore, investigated in this work. The results show that the underlying multi-phase system is already present in the screw section of the extruder. The morphology development mainly takes place in the tapered transition zone and the non-cooled zone, while the cooled zone only has a minor influence. The cross-sectional contraction and the cooling generate elongational flows and tensile stresses in the die section, whereas the highest tensile stresses are generated in the transition zone and are assumed to be the main factor for structure formation. Cooling also has an influence on the velocity gradients and, therefore, the shear stresses; the highest shear stresses are generated towards the die exit. The results further show that morphology development in the die section is mainly governed by deformation and orientation, while the breakup of phases appears to play a minor role. The size of the dispersed phase, i.e., size of individual particles, is presumably determined in the screw section and then stays the same over the die length. Overall, this study reveals that morphology development and flow characteristics need to be understood and controlled for a successful product design in HME, which, in turn, could be achieved by a targeted design of the extruders die section.

## 1. Introduction

Plant-based meat analogues are becoming increasingly popular [1]. The high moisture extrusion (HME) process can be used for their production: plant-based, protein-rich raw materials are processed at water contents above 40%, resulting in a meat-like structure and texture [2,3,4,5]. In the screw section of the extruder, the raw material is mixed with water and subjected to thermomechanical stresses by the screw rotation and the barrel heating, resulting in a flowable mass. The conveying action of the screws then presses this mass through a cooling die attached to the extruder, which prevents expansion of the product at the die exit [6]. Consequently, the desired meat-like, anisotropic structure in the product is achieved.

The cooling die, or more generally, the die section, has a very large influence on the final product structure or can even be regarded as a prerequisite for the formation of the characteristic anisotropic structures in HME. Dead-stop experiments, in which the extruder is abruptly stopped and then samples are taken from different points in the extruder, could show that the formation of anisotropic structures takes place at the beginning of or in the die section [7,8]. The large influence of the die section becomes clear when the shape or orientation of the anisotropic structures is considered: these usually exhibit the V-shape of a laminar flow profile [9,10,11,12,13,14,15], which is attributed to the laminar flow (Re < 1) in the die section.

The anisotropic structures in HME were shown to originate from a multi-phase system in the extrudates [11]: according to the theory of Tolstoguzov [16,17], the thermodynamic incompatibility of the involved biopolymers, i.e., proteins and polysaccharides, leads to a phase-separated system, in which the phases differ in their biopolymer composition and local water concentration. Separated phases were found in various systems of highly-concentrated food biopolymers [15,18,19,20,21,22,23], also when only one raw material component was used [10,11,23,24,25].

In general, the morphology of such viscoelastic multi-phase systems has been shown to depend very much on the process conditions, such as temperature [11,20] or screw speed [10]. This is due to the shear and tensile stresses in the process, which can cause deformation, breakup, and coalescence of the phases [26,27]. These stresses are in particular a function of the local flow conditions, e.g., the distribution of shear and elongational flows [27,28], and of the rheological properties, i.e., both the viscous and elastic properties, of all phases involved [27,29,30,31,32]. Thus, for the HME of meat analogues, it can be expected that the flow characteristics and the rheological properties have a major influence on the morphology development and, therefore, on the product structure.

Nevertheless, there are only a few papers focusing on flow characteristics and morphology development in HME. To the best of our knowledge, the flow conditions in the die section of a HME process have only been investigated in one recent study [33]. Based on the hypothesis that phase separation only occurs when the material cools in the die section, it was shown that the process conditions in the die section (e.g., temperature distribution) have a very large influence on the anisotropic structures in the product. Studies on the morphology development in HME are largely limited to the analysis of product morphology, whereby the clear influences of, e.g., temperature [11,24], screw speed [10], or raw material composition [10,15] could be shown. The morphology development in the process, i.e., at different points in the extruder, has only been investigated by a recently published study [7] on the HME of peanut protein biomass. It was shown that a multi-phase system is formed in the process, which has a random disoriented morphology in the screw section and then obtains an oriented morphology in the die section.

Although the mentioned studies demonstrate the importance of flow characteristics and morphology development in HME, the relationship between these two essential parameters remains unclear. The aim of this study is to clarify the role of both parameters and to investigate the relationship of those in a model HME process for the production of meat analogues.

For the determination of the flow characteristics in the die section, a numerical flow simulation methodology is applied, which has already been used in previous works for the simulation of the screw section [34,35,36,37]. The rheological properties of the material, which are important input parameters for the simulation [38,39], are determined using a closed cavity rheometer [40,41,42,43]. Since the multi-phase morphology is to be analysed throughout the entire die section, the cryo-imaging methodology established by Wittek et al. [11] is extended: samples are taken from the die section in a dead-stop experiment and examined by cryo-imaging, allowing an analysis of the morphology development along the entire die length, thus providing an extensive and spatially resolved link to the flow characteristics. A HME process with soy protein isolate (SPI), in which the formation of anisotropic structures and a multi-phase system has already been demonstrated [11], serves as the basis for the investigations. Temperature dependence of the anisotropic structures was demonstrated there as well and will be the exemplary starting point for this work. At different material temperatures, die pressure, product structure, and morphology will be analyzed. Morphology development and flow characteristics will then be analyzed at two selected process conditions.

## 2. Materials and Methods

### 2.1. Material

In this work, commercial soy protein isolate “Supro ST” from Solae LLC (St. Louis, MO, USA) was used. A protein content of at least 90% on a dry basis is specified by the manufacturer. A moisture content of 3.4% (*w*/*w*) was determined gravimetrically.

### 2.2. Extrusion Trials

Extrusion trials were performed with a co-rotating twin-screw extruder (“Process 11”, ThermoFisher Scientific Inc., Waltham, MA, USA) with a length to diameter (L/D) ratio of 40 and a screw diameter of 11 mm. A slit cooling die of 125 mm length was attached to the extruder through a die adapter, and both were separated by a thin PTFE isolation layer. Only forward elements were used in the screw configuration.

The extruder barrel consisted of eight elements. The first barrel element could be neither heated nor cooled. The seven other barrel elements could be heated and cooled independently of each other. The die adapter, which was attached to the last barrel element, could only be heated.

Solids were dosed via a gravimetrically controlled feeder (Brabender Technology GmbH, Duisburg, Germany) in the first barrel element. Water was dosed via a peristaltic pump (“Masterflex L/S”, Cole Parmer, Vernon Hills, IL, USA) in the third barrel element. The die adapter, which was 32 mm long, provided a transition from the screw section to the cooling die. Temperature control liquid “Thermal HL60” at −10 °C was used to cool the cooling die (125 × 19 × 4 mm) and was supplied by a water-cooled process circulator “Presto Plus LH 47”, both from Julabo GmbH (Seelbach, Germany). 

The screw speed was kept constant at 600 rpm for all process conditions. Always 0.9 kg/h solid SPI and 1.1 kg/h water (tap water with a conductivity of 584.75 ± 16.78 µS/cm from Karlsruhe, Germany) were added, which equals a moisture content of 56.5% (*w*/*w*).

Four different process conditions were set in the extrusion trials, each of them identified by their material temperature T_Material_: 95, 105, 115, 125 °C. To achieve this specific material temperatures, the temperatures of the barrel elements were specifically adjusted. The temperature profiles for the four process conditions are given in Table 1. 

The material temperature and the die pressure were measured simultaneously in the die adapter, 23 mm after the end of the screw section (see Figure 2 for exact location). Sampling for further analysis of the extrudates was performed once material temperature and the die pressure were constant for at least three minutes. The die pressure was then measured for another three minutes, and the average value was taken. To investigate the anisotropic product structure, the extrudates were torn open immediately after exiting the extruder and and the visible structure was photographed. For further analysis of the morphology, the extrudates were vacuumed immediately after exit, frozen, and stored at −18 °C until further use.

Dead-stop trials were performed with the same process settings as described and with the individual temperature profiles for T_Material_ = 95 °C and T_Material_ = 125 °C (Table 1). After the process responses were constant for at least five minutes, simultaneously, the cooling capacity of the extruder barrels was switched to maximum, and the screws stopped. The cooling die and the die adapter were removed from the extruder as quickly as possible and the extrudate removed from the interior. For 125 °C, the temperatures in the last barrel section were too high. Therefore, the product expanded “back” into the screw section, which also slightly influenced the extrudate in the die section. This part of the extrudate in the die section was discarded, and, therefore, cryo-images from the transition zone from screw to die section were only obtained for 95 °C. For further analysis, the extrudate from the die section was immediately vacuumed, frozen, and stored at −18 °C until further use.

### 2.3. Cryo-Imaging

The morphology of the extrudates and the samples from dead-stop trials was analysed by cutting the frozen samples laterally to the flow direction (side view) with a cryo-microtome from Leica Biosystems GmbH (Nussloch, Germany). The cooling room temperature of this instrument was set to −14 °C, while the temperature of the sample holder was set to −12 °C. To prepare the extrudates for cutting, 4–5 cm long sections were cut out from the extrudate strand. These sections were attached to a sample holder with FSC 22 Frozen Section Media, a sectioning medium from Leica Biosystems GmbH (Nussloch, Germany). This was intended to ensure product stability on the sample holder while sectioning and was tested to have no influence on the microstructure of the product. After attachment, 40-µm sections were cut from the extrudate until smooth cut surfaces were achieved. Immediately after cutting the extrudate in the cryo-microtome, the sections were discarded, and photos of the cut surface were taken with a digital camera and a macro-objective (DMC-GH2, Lumix, Kadoma, CGH2, Lumix, Kadoma, Japan).

### 2.4. Rheological Measurements and Fitting

**Figure 1:**
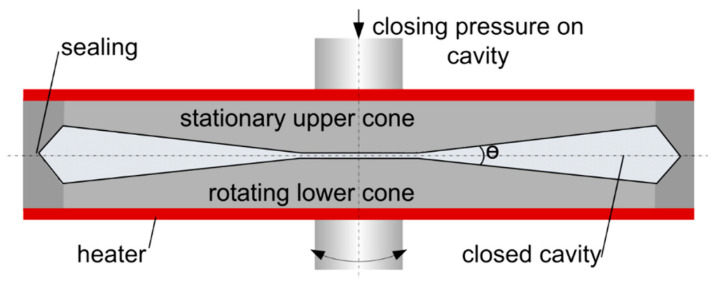
Closed cavity rheometer used for rheological measurements (picture taken from Emin and Schuchmann, 2017 [44]).

To determine the rheological properties for the numerical simulation, samples were analysed in a closed cavity rheometer (CCR) “RPA flex” from TA Instruments, Inc. (New Castle, DE, USA); see Figure 1. This instrument is described in detail elsewhere [40,41] and will only be briefly described here. The material is first placed between the two cones. The downward movement of the upper cone and the application of a closing pressure creates a closed cavity for the material, which prevents water evaporation and allows measurements at high temperatures. The lower cone exerts a sinusoidal rotary deformation, and the resulting force is monitored, from which the resulting rheological properties can be calculated.

For the dough preparation, solid SPI was mixed with deionized water in a Thermomix from Vorwerk (Wuppertal, Germany) to achieve a moisture content of 55% (*w*/*w*), taking into account the raw material moisture. The doughs were then vacuum sealed and stored in a refrigerator at 4 °C for at least 12 h prior to the measurements, which was intended to ensure uniform water distribution and hydration. 

For the measurement, the dough was treated first for 60 s at 125 °C, 240%, and 6.28 rad·s^−1^ (which equals 150 s^−1^). High shear treatment was chosen to imitate thermomechanical stresses in the extruder. It was then quickly cooled to 60 °C, where a frequency sweep at 0.98% was performed. This cooling was necessary, as the motor torque at 125 °C was too low and measurements were not feasible. Since this measurement was done using oscillatory–rotatory measurements, the measured complex viscosity η* was converted to shear viscosity *η* using the Cox-Merz [45] relationship:

$$\left|\eta^{*}\left(\omega \right)\right|=\eta {\left(\dot{\gamma }\right)}_{|\dot{\gamma }=\omega }$$

where η* is the complex viscosity from the oscillatory measurements, ω the corresponding angular frequency, *η* the (shear) viscosity and $\dot{\gamma }$ the shear rate. For the numerical simulation, a viscosity function is necessary that describes the viscosity over the entire range of shear rates. The frequency sweep, however, delivers only data within a limited range of shear rates. Therefore, the rheological data was fitted with a Bird–Carreau [46] viscosity model:

$$\eta \left(\dot{\gamma }\right)=\eta_{\infty }+\left(\eta_{0}-\eta_{\infty }\right)*{\left(1+{\left(\lambda *\dot{\gamma }\right)}^{2}\right)}^{\frac{n-1}{2}})$$

where $\eta \left(\dot{\gamma }\right)$ is the viscosity at a certain shear rate $\dot{\gamma }$, *η*_∞_ is the infinite-shear-rate viscosity, *η*_0_ the zero-shear-rate viscosity, *λ* the natural time, and *n* the power law index. With the assumption of *η_∞_* = 0, the equation simplifies to:

$$\eta \left(\dot{\gamma }\right)=\eta_{0}*{\left(1+{\left(\lambda *\dot{\gamma }\right)}^{2}\right)}^{\frac{n-1}{2}}$$

For high shear rates, this model describes the fluid behaviour as a power-law fluid, while the fluid is described as a Newtonian fluid with a shear-independent viscosity for low shear rates. This model has been suggested before to increase numerical stability in the process [34,35]. The Bird–Carreau model needs the definition of a natural time *λ*, which defines a shear rate at which transition between Newtonian and power-law behaviour takes place. However, as the measurements were not able to detect a viscosity plateau at low shear rates, a value of 100 s was chosen for *λ*, based on values from the literature [47]. As a non-isothermal simulation is aimed for, the temperature dependence of the rheological properties must be determined as well. Therefore, the experimental results from a previous work for the viscosity as a function of temperature (from 50–120 °C) were taken as a basis [11]. The viscosity at a certain shear rate and temperature was factorized as follows:

$$\eta \left(\dot{\gamma },T\right)=k\left(T\right)\cdot \eta \left(\dot{\gamma }\right)$$

where *k*(*T*) is the Arrhenius law constant at a given temperature:

$$k\left(T\right)=\exp\left(\frac{\alpha }{T}-\frac{\alpha }{T_{\alpha }}\right)$$

*α* is the relationship between the activation energy *E_A_* and the universal gas constant R and *T_α_* is a reference temperature for which *k*(*T*) results in a value of 1.

### 2.5. Material Properties for Simulation

The density of the material for the simulation was determined by measuring the outflow velocity of the extrudates at least eight times and multiplying with the cross-section area of the die cross-section, which gives the volume flow (as die swell was negligibly small). Division of the mass flow with the volume flow then gave a density of 1279.55 kg·m^−3^, which was used in the simulation. 

The specific heat capacity *c_p_* was determined with the equation proposed by Singh and Heldman [48]:

$$c_{P}=1.424*X_{c}+1.549*X_{p}+1.675*X_{f}+0.837*X_{a}+4.187*X_{m}$$

where *X* is the mass fraction; the subscripts on the right-hand side are: *c* = carbohydrate, *p* = protein, *f* = fat, *a* = ash, *m* = moisture. This equation gives a specific heat capacity of 3013.9 J·kg^−1^·K^−1^ for the SPI matrix.

The thermal conductivity *k* was calculated with the equation proposed by Sweat [49] that is fitting a set of 430 data points for solid and liquid foods:

$$k=0.25*X_{c}+0.155*X_{p}+0.16*X_{f}+0.135*X_{a}+0.58*X_{m}$$

where *X* is the mass fraction; the subscripts on the right-hand side are: *c* = carbohydrate, *p* = protein, *f* = fat, *a* = ash, *m* = moisture. This equation gives a thermal conductivity of 0.395 J·s^−1^·m^−1^·K^−1^ for the SPI matrix. Thermal expansion, inertia, and gravity were neglected; viscous heating was taken into account.

### 2.6. Numerical Simulation

**Figure 2:**
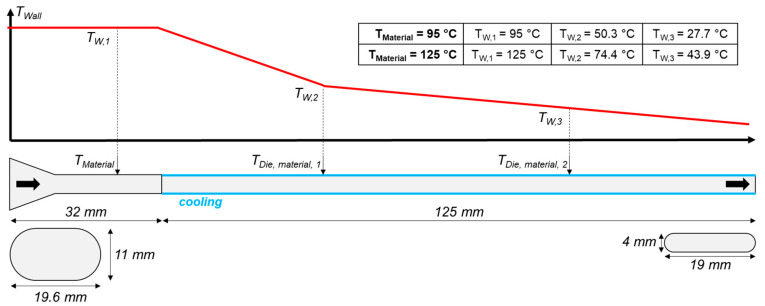
Modelling of the flow domain, which resembles the die section from extrusion trials, and the temperature profile.

The calculation of the numerical equations was performed with ANSYS POLYFLOW^®^ (Version: 2020 R2, Ansys Inc., Canonsburg, PA, USA), a computational fluid dynamics program that has previously been applied for comparable analyses in extrusion processes, mainly focusing on the screw section [34,35,36,37,50]. The simulated domain was modelled to correspond to the real die section from the extrusion trials as close as possible (a three-dimensional overview can be seen in the Appendix A). The modelling parameters are described in Figure 2.

The die section consisted of the die adapter (not cooled) and the cooling die (cooled), with a length of 32 mm and 125 mm, respectively. The die section inlet had a rectangular shape with dimensions of 19.6 × 11 mm and rounded corners. A tapered section then led to a narrower rectangular flow channel with dimensions of 19 × 4 mm, which kept these dimensions until the die outlet. 

The definition of the wall temperatures was based on material temperature measurements in the die section during the extrusion trials (red curve). The location of measurements is displayed (T_Material_, T_Die,Material,1_, and T_Die,Material,2_). Measurements were performed with common thermocouples, which were flush with the material inside the die. It is assumed that this measured temperature is the material temperature at the edge of the die channel, and therefore could be taken for the definition of the wall temperature profile in the simulation. The “temperature imposed” thermal boundary condition was chosen for the walls; the temperature of the fluid entering the domain was defined according to the two investigated process conditions, either 95 or 125 °C. A no-slip condition (zero wall velocity) was defined for the walls, and a mass flow of 2.0 kg/h was defined for the inlet. 

Tensile stresses were computed according to the equation [51]:

$$\sigma_{E}=\eta_{E}\;\cdot \sqrt{{\frac{\partial {\overset{-}{v}}_{x}}{\partial x}}^{2}+{\frac{\partial {\overset{-}{v}}_{y}}{\partial y}}^{2}+{\frac{\partial {\overset{-}{v}}_{z}}{\partial z}}^{2}}$$

where *η_E_* is the elongational viscosity and *v_x_*, *v_y_*, *v_z_* correspond to the velocities in x-, y-, and z-direction. The elongational viscosity *η_E_* (also known as extensional viscosity) is defined according to the “Trouton ratio” [52] *η_E_* = 3·*η*, where *η* is the shear viscosity. For viscoelastic fluids, this equation will hold in the limiting case of low elongational rates [53].

**Figure A1:**
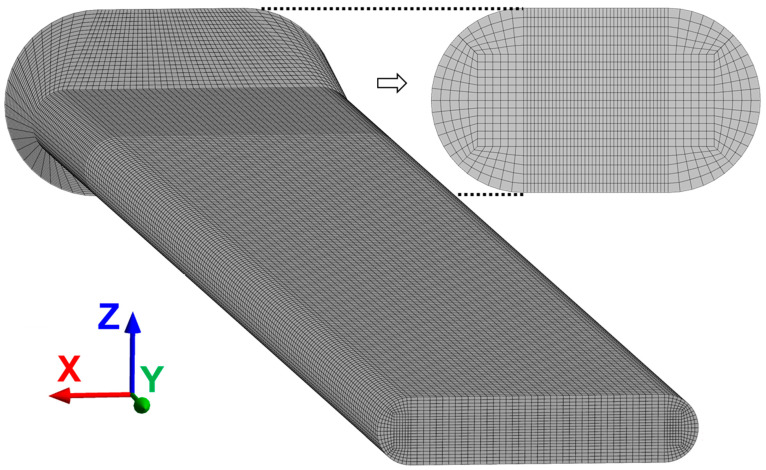
Hexahedral mesh with 69,520 elements used for the numerical simulations.

Mini-elements for velocities and linear pressure were chosen for interpolation settings. Iterations on the viscosity were performed with a Picard scheme. The temperature field was solved with the setting “2 × 2 element for temperature” in order to get a locally higher resolution of the temperature field and improve solution accuracy. Upwinding in the energy equation was activated. After performing sensitivity analysis by investigating the influence of mesh design and interpolation method on the velocity profile in the die, a regular hexahedral mesh with 69,520 elements was chosen for the simulations, see Figure A1 in the Appendix A for a 3D illustration.

## 3. Results

### 3.1. Influence of Material Temperature on Die Pressure, Product Structure, and Morphology 

**Figure 3:**
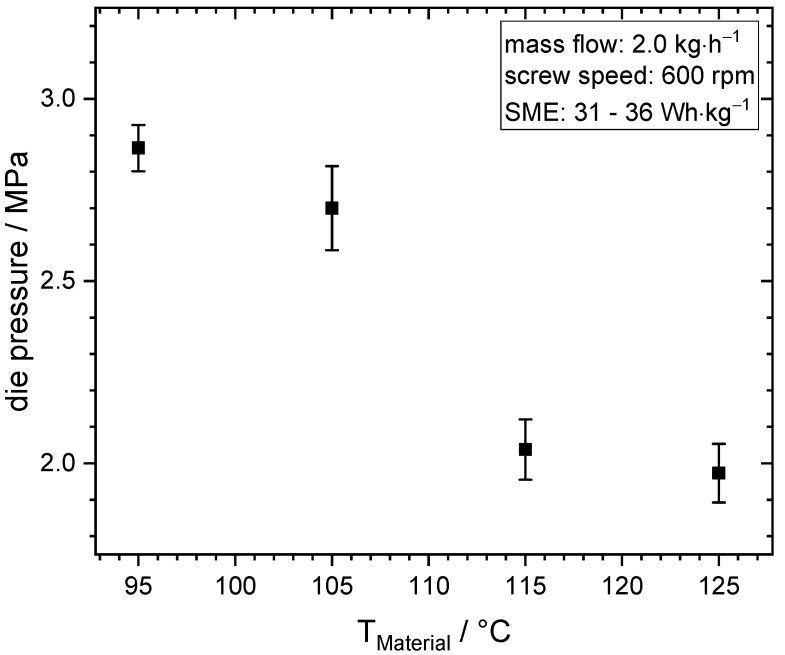
Die pressure as a function of material temperature at constant mass flow (2.0 kg·h^−1^) and screw speed (600 rpm).

Extrusion trials at different material temperatures served as a basis for further investigations of morphology development and flow characteristics. The die pressures from the extrusion trials are plotted as a function of material temperature in Figure 3.

**Figure 4:**
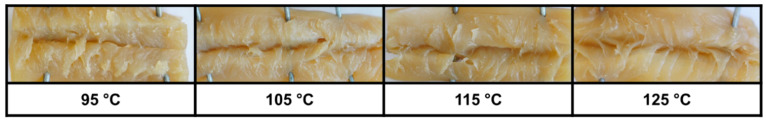
Product structure of the extrudates at the four different investigated material temperatures.

As the material temperature increased from 95 to 125 °C, the die pressure decreased from 2.9 to 2.0 MPa. The die pressure is a function of the material viscosity at constant mass flow and die geometry. Thus, the results indicate that viscosity decreased with increasing material temperature. Since it is expected that covalent protein–protein interactions are neither formed nor broken at the present process conditions in the soy proteins [11], and therefore the molecular weight is not effected, the decreased viscosity can be attributed to increased mobility of the molecules increasing temperature [54]. The product structure of the extrudates is displayed in Figure 4.

**Figure 5:**
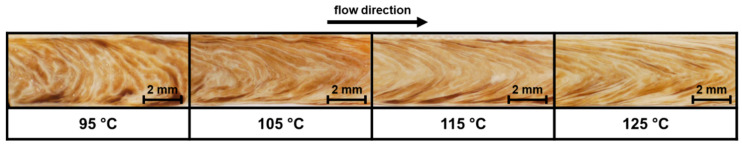
Morphology of the extrudates at the four different material temperatures obtained via cryo-imaging. Flow direction is from left to right; lateral view displayed.

With increasing material temperature, a more pronounced anisotropic structure was apparent in the extrudates. At 95 °C, the structure was still dough-like, while at 125 °C, more pronounced structures were visible. An influence of temperature on the product structure could be observed for various other material systems in HME as well [11,14,15,24,55,56]: the temperature increase also resulted in more pronounced structures or led to improved textural properties (e.g., higher anisotropy index). The morphology of the extrudates was studied by cryo-imaging and is displayed in Figure 5.

A multi-phase system, in which an elongated dispersed phase is distributed in a continuous phase, was formed at all material temperatures. The formation of a multi-phase system can be explained by the thermodynamic incompatibility of the involved proteins [16], which leads to a phase separation and phases that differ in their local biopolymer composition and water concentration [11,18,21,57]. The increase in material temperature influences the morphology of the multi-phase system. The V-shape, which originates from the laminar flow profile in the die, was only very weakly pronounced for 95 °C and rather flat. It became more pointed with increasing temperature, while at the same time, the size and shape of the dispersed phase also changed. The individual particles/elements of the dispersed phase appeared shorter and more compact at 95 °C compared to 125 °C, where they were longer and more elongated. It is assumed that the changes in morphology development are the reason for the different product structures and were caused by the decreasing viscosity as indicated by the die pressure. For example, decreasing viscosity could have led to lower shear stresses in the process. However, the influence of material temperature on morphology development and flow characteristics should be determined in detail in the next chapters.

### 3.2. Rheological Data 

**Figure 6:**
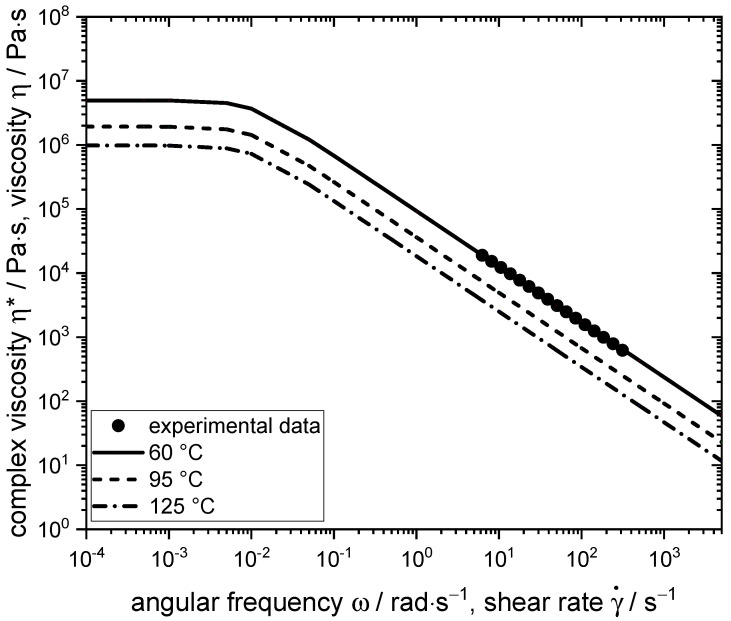
Experimentally determined complex viscosity as a function of the angular frequency at 60 °C (filled circles) and the fitted viscosity curves at 60, 95, and 125 °C, which were based on the Bird–Carreau model.

Process-relevant rheological properties of the material are required for the numerical simulation and were determined using a CCR. The resulting flow curves are plotted in Figure 6, with exemplary curves shown for 60 °C, where the frequency sweep was measured, and for 95 °C and 125 °C to illustrate the temperature dependence.

The material exhibited shear-thinning behaviour, which is typical for highly concentrated SPI [11,40,58,59,60,61,62]. This behaviour is expected to have an influence on the morphology development: in a shear-thinning matrix, deformation and/or breakup of the dispersed phase is more difficult compared to a Newtonian matrix because the increase in viscous stresses is smaller for the same increase in shear rate [63]. The parameters of the fitted flow curve at 60 °C, which was based on the Bird–Carreau model, and the parameters for the temperature dependence from the Arrhenius model are listed in Table 2.

The activation energy E_A_ of the material system can be determined from the parameter α and calculates as 27.55 kJ·mol^−1^. This is in the range of what is reported for other (bio-)polymers [54,64]. 

### 3.3. Morphology Development in the Die Section and Influence of Material Temperature

**Figure 7:**
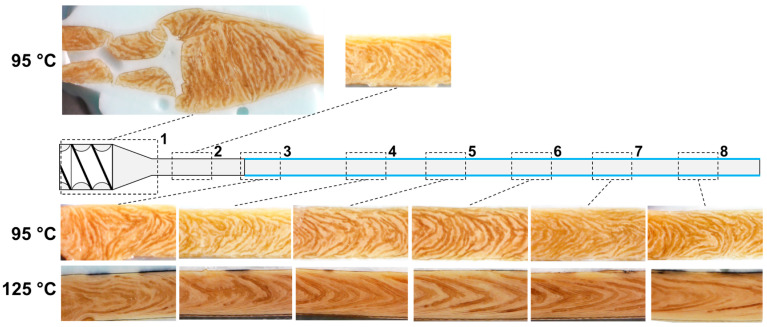
Morphology of samples obtained through cryo-imaging in dead-stop trials at T_Material_ = 95 °C and 125 °C from different locations in the die section. Flow direction is from left to right.

In order to study the morphology development throughout the die section, dead-stop extrusion trials were performed at two selected process conditions, i.e., at material temperatures of 95 °C and 125 °C, and samples were taken from the die section in each case. The results are shown in Figure 7. From the first two sections, i.e., the tapered transition zone (section 1) and the non-cooled zone (section 2), it was only possible to take a sample at 95 °C (top row). 

Analog to the previous findings from extrusion trials (Figure 5), a multi-phase system was present at both material temperatures. The individual particles of the dispersed phase also appeared shorter and more compact at 95 °C compared to 125 °C, where they were longer and more elongated. This multi-phase system was present along the entire length of the die section, including the screw section, for both temperatures. It can thus be assumed that as soon as the plastification of material in the screw section allows the mobility of the protein molecules, the thermodynamic incompatibility of the proteins practically immediately leads to phase separation in the material. 

At the beginning of the tapered transition zone between the screw section and the die section (section 1), the V-shape was not apparent yet; instead, the dispersed phase was oriented transversely to the flow direction. However, a V-shape began to form in the direction of flow and was retained in the uncooled zone (section 2). From the third section onwards, where the start of cooling was marked with the continuous transverse line and the blue lines at the edges, cryo-images were available for both material temperatures (lower two rows of images). In section 3 at 95 °C, the phases were only slightly deformed in the left part of the image, while in the right part, they showed a much more pronounced V-shape. Further, in the direction of flow, this deformation or V-shape was then largely preserved, and the morphology did not undergo major changes. A V-shape morphology was also already visible from the third section at 125 °C. However, the shape was more pronounced, i.e., more pointed, than at 95 °C. In the flow direction and in contrast to the lower temperature, the V-shape developed slightly further, i.e., the shape became more pointed and the dispersed phase more elongated. This slight development of the morphology continued until the last section, where the dispersed phase was the most elongated. In the last section (8), i.e., right before the die exit, the 125 °C sample exhibited a much more pronounced V-shape and more elongated dispersed phase than the 95 °C sample. 

The results showed that the multi-phase system was already present in the screw section and thus that the morphology was developing along the entire die section, i.e., in the tapered transition zone, uncooled zone, and cooled zone. For 95 °C, the V-shape was already developed right after the transition zone (sections 1 and 2), and for 125 °C, the V-shape was already developed at the beginning of the cooled zone (section 3). This matches the findings from other studies, where the formation of anisotropic structures has been observed in the transition zone of the die section (i.e., right after the screw section) [7,8].

It also appears that the influence of each die zone on the morphology development was different: for instance, morphology development seems to be more pronounced in the transition zone compared to the cooled zone. A possible explanation for this could be the differences in geometrical dimensions (tapered vs. non-tapered) and thermal conditions (cooled vs. non-cooled), which would be expected to largely influence the local flow characteristics. However, the exact relationships remain unclear, and the investigation of flow characteristics in the next chapter should clarify how morphology development is influenced in the die section.

### 3.4. Flow Characteristics in the Die Section and Influence of Material Temperature

**Figure 8:**
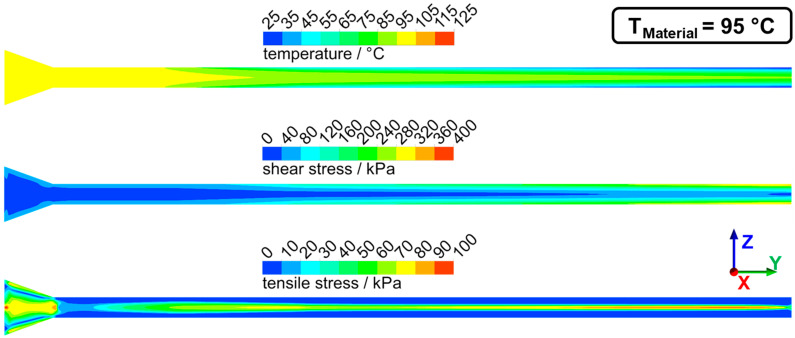
Temperature, shear stress, and tensile stress distribution in the die section of extrusion trials with T_Material_ = 95 °C. The lateral view is displayed, flow direction from left to right (in the y-direction).

**Figure 9:**
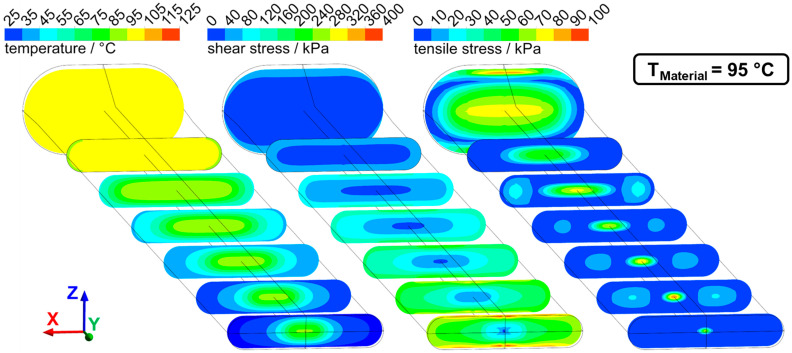
Temperature, shear stress, and tensile stress distribution in the die section at T_Material_ = 95 °C in sectional views transversely to the flow direction.

**Figure 10:**
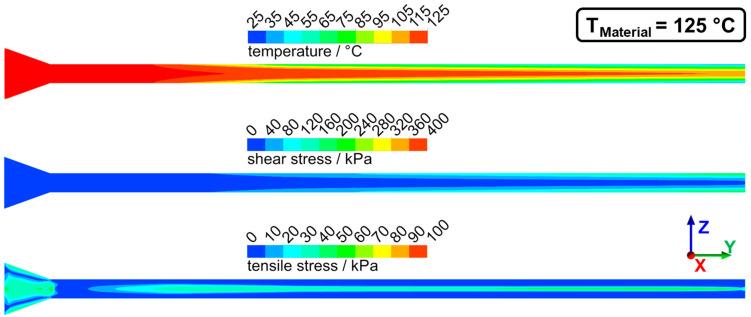
Temperature, shear stress, and tensile stress distribution in the die section of extrusion trials with T_Material_ = 125 °C. The lateral view is displayed, flow direction from left to right (in the y-direction).

**Figure 11:**
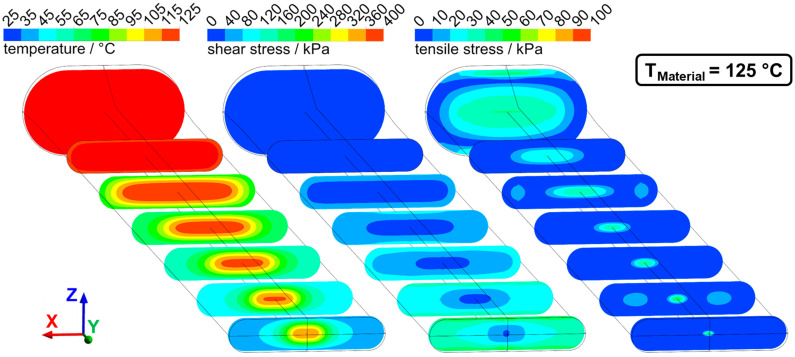
Temperature, shear stress, and tensile stress distribution in the die section at T_Material_ = 125 °C in sectional views transversely to the flow direction.

Numerical simulation was used to determine the flow characteristics in the die section. Temperature, shear stresses, and tensile stresses were determined for the previously discussed two cases of dead-stop at 95 °C and 125 °C. The distributions of these parameters from two perspectives (lateral and transversal view) can be seen for 95 °C in Figure 8 (lateral view) and Figure 9 (transversal view) and for 125 °C in Figure 10 (lateral view) and Figure 11 (transversal view). To ensure comparability, the scales of the individual parameters are kept the same for the different views and temperatures.

The die section of the extruder consisted of a die adapter (front part) and the actual cooling die (rear part). Not the entire die section was cooled: cooling of the material only begins in the cooling die, as can be seen from the temperature distribution. The material cools from the outside to the inside, and at the die outlet, there is a temperature gradient from the warm core to the cold exterior. The material core reached a temperature of 102 °C for T_Material_ = 125 °C and a temperature of 70 °C for the case of T_Material_ = 95 °C. Such a relatively large temperature gradient between core and exterior could be confirmed in the extrusion trials, where an intruding temperature couple was used to determine the extrudates core temperature right after exiting the extruder.

**Figure 12:**
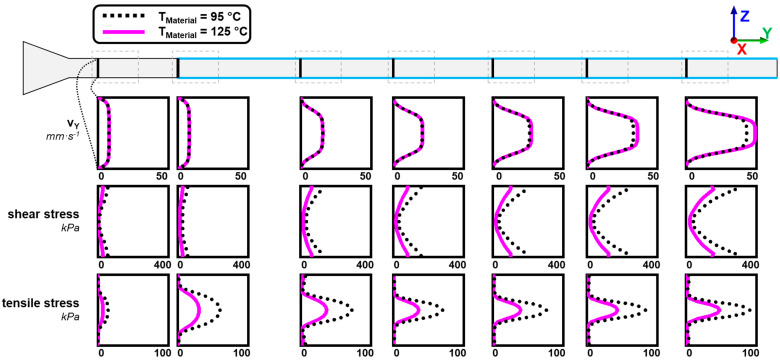
Development of profiles of velocities in the flow direction, shear stresses, and tensile stresses along the die length for both material temperatures 95 °C (black dotted line) and 125 °C (pink solid line). Locations of profile analysis (black solid lines transversely to flow direction) correspond to the location of cryo-sections from the dead-stop experiment (Figure 7), which are marked with the grey dashed lines.

The temperature distribution over the cross-section also resulted in a distribution of viscosity: a higher viscosity was achieved in the cool exterior than in the core. This had a very large influence on the velocity profiles in the process, as can be seen in Figure 12. The velocity of the fluid in the flow direction is plotted for different locations along the die as a function of height coordinate z (thus corresponding to a lateral view).

While a relatively flat velocity profile can be seen in the first profiles, a much more curved velocity profile developed along the die length due to the cooling, with higher flow velocities in the core. Higher maximum core velocities were reached at 125 °C (49 mm·s^−1^) than at 95 °C (43 mm·s^−1^).

This influence of the temperature distribution on the flow velocities had two effects: on the one hand, the high gradients towards the edge resulted in high shear rates and therefore high shear stresses, and on the other hand, the fluid acceleration in the core generated an elongational flow and therefore tensile stresses. Both had an influence on the distribution of shear and tensile stresses in the process. The shear stresses increased along the die length. This can be explained by the higher shear rates due to the higher velocity gradients and by the overall increasing viscosity as a result of cooling. The highest shear stresses (up to 400 kPa at 95 °C) were generated at the die outlet and the die edges, while the shear stresses in the upstream part were much lower (less than 100 kPa at 95 °C). 

Elongational flows were generated at two points in the die section: first in the tapered transition zone and then by the acceleration of the flow in the cooled zone. This can be seen in the distribution of tensile stresses. High tensile stresses were generated in the transition zone (up to 100 kPa at 95 °C) over almost the entire cross-section. These tensile stresses then decreased down to less than 20 kPa in the non-cooled zone. In the cooled zone, tensile stresses again increased in the core (up to 90 kPa at 95 °C). Towards the end, the tensile stresses then decreased again.

The three characteristic parts of the die section, i.e., the tapered transition zone, the non-cooled zone, and the cooled zone, can, therefore, be differentiated by their specific flow conditions. In the transition zone, the highest tensile stresses (up to 100 kPa) and relatively low shear stresses (not more than 80 kPa) were generated. The non-cooled zone had medium-high tensile stress (up to 50 kPa at 95 °C) and also relatively low shear stresses (not more than 80 kPa). In the cooled zone, the highest shear stresses (up to 400 kPa) and relatively high tensile stresses (up to 80 kPa) were generated.

These findings on the flow characteristics could be helpful to understand the morphology development in the die section. The deformation and orientation of the dispersed phase at 95 °C already started in the transition zone (section 1 in Figure 7), and it is assumed that this would also be the case at 125 °C. The transition zone was dominated by the highest tensile stresses in the die section (up to 100 kPa at 95 °C), while the shear stresses were comparatively low (less than 80 kPa at 95 °C). It is, therefore, assumed that the tensile stresses have an important role in the deformation and orientation of the dispersed phase. The importance of the tensile stresses became apparent in section 3 (Figure 7) at 95 °C: with the onset of cooling in this section, the dispersed phase also underwent a very strong deformation, which is expected to result from the generated tensile stresses due to the elongational flow at this point; similar to the transition zone, the shear stresses were also comparatively low at this point (less than 80 kPa). It is known that tensile stresses are much more effective in deforming and/or breaking up the dispersed phase in viscoelastic matrices [27,28]. For example, elongational flows enable deformation/breakup at high viscosity ratios where simple shear flows would not. The effectiveness of tensile stresses compared to shear stresses can also be seen in a comparison of extruder and shear cells, which operated at similar temperatures and water contents: in the shear cell, where almost exclusively shear stresses occurred, a significantly longer process time of up to 15 min [65] was required to obtain structures comparable to those in the extrusion process, where a mix of shear and tensile stresses was present and the process time/residence time was less than two minutes. 

The morphology development in the cooled zone was lower than in the transition and non-cooled zone (Figure 7). At 95 °C, the morphology at the beginning of the cooled zone was almost the same as at the end. It appeared that the increase in shear stress over the die length did not influence the morphology. 

In a comparison of the flow characteristics between 95 °C and 125 °C, temperature, shear stress, and tensile stress showed similar distributions but different absolute values. The overall higher temperatures in the die section were caused by the different defined material temperatures at the die inlet. Significantly higher shear and tensile stresses were obtained at 95 °C compared to 125 °C; maximum shear and tensile stresses were almost twice as high at 95 °C. Both were a function of the viscosity, which decreased with increasing temperature and therefore resulted in lower overall stress values (as the flow profiles are very similar). It would therefore be expected that deformation and orientation of the dispersed phase are more pronounced at 95 °C, as higher absolute stress values were apparent here. But the opposite was the case: for 125 °C, which had lower stresses, deformation and orientation were more pronounced. Thus, the simple coherence between viscosity and shear stresses in the die section cannot explain the differences between the temperatures. 

It appears that only deformation and orientation of the dispersed phase take place in the die section. The size of the dispersed phase, i.e., the size (or surface area) of individual particles, seems to remain roughly the same. The differences in dispersed phase size between both temperatures were already established in the non-cooled zone. It is therefore assumed that the screw section also has an important influence on the morphology development by determining the morphology in which the die section is entered. Different explanations for the temperature influence could then be possible. Due to the higher viscosity at low temperature, the stresses in the screw section were also higher, and thus, the breakup of the dispersed phase could be favoured, which could explain the apparently smaller dispersed phase. However, the breakup of phases does not only depend on the viscosity of the continuous phase but also on the viscosity ratio of the phases [31]. It has also been shown that the viscosity ratio has a large influence on the shape of the dispersed phase; elongated shapes could only be achieved when the viscosity of the dispersed phase was lower than the continuous phase [31]. The viscosity ratio could also be temperature-dependent, e.g., when the rheological properties of the phases have different temperature dependencies [28], and it, therefore, remains unclear how morphology development is actually influenced. Furthermore, in addition to breakup, coalescence in the screw section should not be neglected [26]: this could also have been influenced by temperature and thus explain the different sizes of the dispersed phases.

## 4. Conclusions

The variation of material temperature in a HME process with SPI, had an influence on the die pressure, the product structure, and the morphology of the extrudates. The temperature increase resulted in lower die pressure, more pronounced anisotropic structures, and a more deformed shape of the dispersed phase, i.e., a more pointed V-shape. Analysis of the morphology development for two selected material temperatures throughout the die section revealed that the underlying multi-phase system is already present in the screw section. The size of the dispersed phase, i.e., size of individual particles, appears to be not subjected to changes in the die section; instead, the morphology is mainly influenced through deformation and orientation. The morphology development occurred mainly in the tapered transition zone and the non-cooled zone, while only minor changes occurred in the cooled zone. Tensile stresses of up to 100 kPa were generated in the die section, which can be attributed to the generation of elongational flow due to the cross-sectional contraction in the transition zone and acceleration of fluid caused by cooling, respectively. Comparison of the two simulated process conditions revealed that the screw section must also have a large influence on the morphology by setting the size of the particles of the dispersed phase when entering the die section.

Overall, the results of this study suggest that the flow characteristics have a large influence on morphology development; especially the tensile stresses are assumed to play a major role. The cross-sectional contraction in the transition zone and the cooling are responsible for the generation of elongational flows and thus the tensile stresses. These die parameters, specifically the geometrical dimensions of contraction and the die temperature, should be considered in future process design. Understanding the flow characteristics could be helpful in adapting the morphology development to the product design requirements in HME.

## Figures and Tables

**Table 1:** Temperature profiles of the extrusion trials at the four different material temperatures. Dead-stop trials were performed with the same specific temperature profiles at 95 °C and 125 °C.

T_Material_/°C	95	105	115	125
T_Barrel,2_/°C	25	25	25	25
T_Barrel,3_/°C	50	50	50	50
T_Barrel,4_/°C	90	90	90	90
T_Barrel,5_/°C	98	109	110	110
T_Barrel,6_/°C	98	109	124	138
T_Barrel,7_/°C	98	109	124	138
T_Barrel,8_/°C	98	109	124	138
T_Die_/°C	98	109	124	138

**Table 2:** Parameters of the Bird–Carreau viscosity curve at 60 °C and the parameters from Arrhenius law to describe temperature dependence.

*η*_0_/Pa·s	*λ*/s	*n*/-	*α*/*K*	*T_α_*/*K*
4,952,779	100	0.13606	3313.3	333.15
